# Characterisation of the Expression of NMDA Receptors in Human Astrocytes

**DOI:** 10.1371/journal.pone.0014123

**Published:** 2010-11-30

**Authors:** Ming-Chak Lee, Ka Ka Ting, Seray Adams, Bruce J. Brew, Roger Chung, Gilles J. Guillemin

**Affiliations:** 1 Department of Pharmacology, School of Medical Sciences, University of New South Wales, Sydney, Australia; 2 St. Vincent's Centre for Applied Medical Research, St. Vincent's Hospital, Sydney, Australia; 3 Departments of Neurology and HIV Medicine, St. Vincent's Hospital, Sydney, Australia; 4 NeuroRepair Group, Menzies Research Institute, University of Tasmania, Hobart, Tasmania, Australia; University of Melbourne, Australia

## Abstract

Astrocytes have long been perceived only as structural and supporting cells within the central nervous system (CNS). However, the discovery that these glial cells may potentially express receptors capable of responding to endogenous neurotransmitters has resulted in the need to reassess astrocytic physiology. The aim of the current study was to characterise the expression of NMDA receptors (NMDARs) in primary human astrocytes, and investigate their response to physiological and excitotoxic concentrations of the known endogenous NMDAR agonists, glutamate and quinolinic acid (QUIN). Primary cultures of human astrocytes were used to examine expression of these receptors at the mRNA level using RT-PCR and qPCR, and at the protein level using immunocytochemistry. The functionality role of the receptors was assessed using intracellular calcium influx experiments and measuring extracellular lactate dehydrogenase (LDH) activity in primary cultures of human astrocytes treated with glutamate and QUIN. We found that all seven currently known NMDAR subunits (NR1, NR2A, NR2B, NR2C, NR2D, NR3A and NR3B) are expressed in astrocytes, but at different levels. Calcium influx studies revealed that both glutamate and QUIN could activate astrocytic NMDARs, which stimulates Ca^2+^ influx into the cell and can result in dysfunction and death of astrocytes. Our data also show that the NMDAR ion channel blockers, MK801, and memantine can attenuate glutamate and QUIN mediated cell excitotoxicity. This suggests that the mechanism of glutamate and QUIN gliotoxicity is at least partially mediated by excessive stimulation of NMDARs. The present study is the first to provide definitive evidence for the existence of functional NMDAR expression in human primary astrocytes. This discovery has significant implications for redefining the cellular interaction between glia and neurons in both physiological processes and pathological conditions.

## Introduction

N-methyl D-aspartate (NMDA) receptors (NMDARs) are ligand-gated ion channels which form one group of ionotropic glutamate receptors in the CNS. These receptors are known to be expressed in neurons and are activated by neurotransmitters including glutamate and NMDA [1] as well as endogenous excitotoxins such as quinolinic acid [2]. NMDARs exist as heterotetrameric complexes at the surface membrane. Currently, seven known subunits have been identified: one NR1, four NR2 (A–D) and two NR3 (A–B) subunits. In neurons, NMDARs play an important role in facilitating learning and memory [3]. However, recent studies have revealed that these membrane proteins may also exist in other cell types [4], [5], [6], [7].

Although research into astrocyte NMDARs is still controversial and functional expression of these receptors in humans is yet to be confirmed [8], [9], there has been some evidence from animal models suggesting the involvement of astrocytic glutamate receptors in glial cell signalling [4], [10], [11], [12], [13]. This form of glial communication involves the induction of an intracellular calcium wave, which was first elicited through the stimulation of cultured astrocytes with glutamate [14]. The study showed that astrocytes could in fact respond to extracellular neurotransmitters such as those released by neurons via a signalling pathway that could potentially be used for physiological glial communication although more research is required to elucidate the exact pathways of activity.

The astrocytic glutamatergic system has also been implicated in several neuropathological conditions including amyotrophic lateral sclerosis (ALS) [15] and Alzheimer's disease (AD)[16]. We have previously shown that extracellular levels of the NMDAR agonist and neurotoxin quinolinic acid (QUIN) are significantly increased in both AD and ALS [17], [18]. QUIN is an endogenous metabolite of L-tryptophan, which is produced via the kynurenine pathway [19]. In the brain, the amino acid L-tryptophan is normally used in protein synthesis and metabolised to compounds such as 5-hydroxytryptamine and other indoleamines. However, during neuroinflammatory conditions, increased activity of the enzyme, indoleamine-2,3-dioxygenase (IDO-1) directs metabolism down the kynurenine pathway and increases the formation of QUIN [20]. Following immune activation in the brain, QUIN is produced by activated microglia and invading macrophages, with high levels of this neurotoxin associated with increased neuronal [21] and glial cell death [19]. These changes are seen in a number of diseases including AD [17] and AIDS dementia complex [22]. As QUIN is an NMDAR agonist, the activation of glutamatergic pathway is likely to be responsible for the cytotoxic effects observed in astrocytes [23], [24].

The aim of this study was to investigate the expression of NMDARs in human primary astrocytes as well as characterise the response of these receptors to physiological and excitotoxic concentrations of known NMDAR agonists. Confirming whether NMDARs exist in astrocytes and examining the function of these receptors in glial cells will further enhance our understanding of the functional role astrocytes play in normal, healthy environments, as well as their potential involvement in neuropathological conditions [25], [26].

## Materials and Methods

### Reagents and chemicals

Dulbecco's phosphate buffered saline (PBS) 1×, RPMI medium 1640 1×, 0.5% Trypsin-EDTA 10×, Glutamax-1 100×, Antibiotic-Antimycotic (AA) 100×, Trizol were obtained from GIBCO Invitrogen (Victoria, Australia). Glucose intravenous infusion BP 50% was from AstraZeneca (Sydney, Australia). Foetal calf serum (FCS) was obtained from Bovogen Australia. Culture flasks and plates were purchased from Becton Dickinson lab ware (New Jersey, USA). QUIN and glutamate were obtained from Sigma Chemical (Sydney, Australia). PCR reaction buffer 10×, MgCl_2_, dNTP and Taq DNA polymerase were obtained from Roche (Mannheim, Germany). Permanox chamber-slides were obtained from Lab-Tek (California, USA) and Fluoromount-G was obtained from Southern Biotech (Alabama, USA.). The BD Calcium Assay Kit was obtained from BD Biosciences (California, USA) and the Fluostar Optima Fluorometer was obtained from BMG LABTECH (Victoria, Australia). The Hepes-buffered Krebs solution, Fura-2 and pluronic F-127, glutamate, QUIN, MK-801, and memantine were purchased from Sigma-Aldrich (Missouri, USA). Trans-ACBD was obtained from Tocris Biosciences (Bristol, UK). The Microplate reader 680XR was obtained from Bio-Rad (California, USA).

### Cell culture

#### Human ethics approval

Human adult brain tissue and human foetal brain tissue were obtained following informed written consent. This has been respectively approved by the Human Ethics Committees from St Vincent's Hospital (HREC 08284) and from the University of New South Wales (UNSW Ethic approval HREC 03187).

#### Isolation and culture of primary astrocytes

Human brain tissue was obtained from adult donors post-surgery and 16- to 19-week-old foetuses. Astrocytes were prepared using a previously described protocol [27]. Briefly, cerebral portions were washed thoroughly with PBS and dissociated by repeated pipettings. The suspension was centrifuged at 500 g for 5 minutes and the cell pellet resuspended in RPMI 1640 medium containing 10% heat-inactivated FCS, 1% glutamax-1, 1% antibiotic-antimicrobial liquid, 0.5% glucose, then plated onto 75 cm**^2^** culture flasks and incubated at 37°C. Medium was changed on the 3^rd^, 5^th^ and 10^th^ day. The cells became confluent after 10–12 days. Microglia were detached from the cultures by mechanically shaking the flasks for 2 hours at 220 rpm at room temperature and aspirated. The astrocytes were trypsinized and replated at least three times to further purify and isolate astrocytes from contaminating microglia and neurons. Astrocytes were left to recover for 3 days after each passage. The astrocytes were rinsed twice with PBS and cultured as above in uncoated flasks with the culture medium and maintained for up to 6 weeks. For the all the following experiments, we have used cell cultures at 3–4 weeks. The medium was changed twice a week. Culture purity was determined by immunofluorescence analysis with antibodies against GFAP and more than 95% of the cells stained positive for GFAP (Figure S1). We did not detect any staining for CD68 (microglia) and for 5B5 (fibroblasts); data not shown [**28**].

#### Isolation and culture of primary neurons

Using the same brain samples mentioned above, human primary neurons have been isolated and grown as we have previously described [29].

### End-point RT-PCR

RNA extraction was undertaken using the Invitrogen Trizol protocol. The cDNA was subsequently quantified via spectrophotometry. Original primers for the seven NMDAR subunits were designed using ‘Primer3’ primer design software, then tested and optimized for the PCR. The individual primers were analysed *via* the BLAST database to ensure specificity of binding. Refer to Table 1 for list of primers. For each NMDAR subunit being tested, 1 µl of cDNA sample (1 µg/µl) was added to a PCR reaction mixture of 49 µl containing: 10 µl of PCR reaction buffer 10×, 5 µl of 2.5 mM MgCl_2_, 2 µl of 10 mM dNTP, 1 µl of 25 mM forward primer, 1 µl of 25 mM backward primer, 0.5 µl of 5 U/µl Taq DNA polymerase and 29.5 µl of DEPC water. PCR was subsequently run on samples for 40 cycles under the following conditions: denaturation (94°C for 60 s), annealing (60°C for 60 s) and extension (72°C for 90 s). Positive controls for primers of the NMDAR subunits involved using reverse transcribed cDNA from a mixture of neuronal and glial brain cells.

**Table 1:** Summary of Primers and product size.

END-POINT PCR			
Human Target cDNA	Accession no.	Forward Primer (5′–3′)	Reverse Primer (5′–3′)	Amplicon (bp)
**NR1**	NM000832	CAAGTATGCGGATGGGGTGA	CAGTCTGGTGGACATCTGGTA	211
**NR2A**	NM000833	CCCCAAACTCCTCAAATCAA	CAGGCGACTCAGAAATGACA	206
**NR2B**	NM000834	ATTGGTGGCAGAGTGGATTC	GGCAAAAGAATCATGGCTGT	463
**NR2C**	NM000835	GACGAGATCAGCAGGGTAGC	ATGGCCAGGATTTCATGGTA	201
**NR2D**	NM000836	AATAATTCGGTGCCCGTGGA	CCCAGACACAGTATCCACGTA	153
**NR3A**	NM133445	GCTTGGGCATCTTAGTGAGG	TACCATGACAGCAGCCAAGT	353
**NR3B**	NM138690	TCCTACTCCTCAGCCCTCAA	ATGTCGGGGAAGCTCTTCTT	296
**REAL-TIME PCR**				
**NR2A**	NM000833.3	GGGCTGGGACATGCAGAAT	CGTCTTTGGAACAGTAGAGCAA	116
**NR2B**	NM000834.3	TTCCGTAATGCTCAACATCATGG	TGCTGCGGATCTTGTTTACAAA	104
**NR2C**	NM000835.3	GCTGGAAGAGCGGCCCTTTGT	CGCTGCTGAAGGTGTGGTTGCTCT	110
**NR2D**	NM000836.2	CTGCAGCCAGTGGACGACACG	GGGTTCGGTTGAGCTGGCTCCG	142
**NR3A**	NM133445.2	GCCACTCCACTGGACAATGTGGC	TTCGCCCCTTGGGAGTCAAACCA	113
**GAPDH**	NM002046	TGCACCACCAACTGCTTAGC	GGCATGGACTGTGGTCATGAG	87

### Real-Time PCR

Human foetal astrocytes prepared from 17 to 20-week-old foetuses (n = 4) were used for real-time PCR. Human foetal neurons were used for primer optimisation and as a positive control for target gene expression. Primer optimisation was performed using a standard curve derived from serial dilutions of human foetal neuron cDNA (0.5 pg–50 ng). Multiple primer pairs for each gene were tested and the most suitable primer candidates were chosen by assessing the specificity of the PCR product and efficiency. Specificity was verified by a single peak in melting curve analysis. The real-time PCR assay for unknown samples was performed simultaneously with positive control samples (neurons) in the same plate.

Total RNA was extracted using the PureLink RNA Mini Kit according to the manufacturer's protocol. RNA quantity was evaluated spectrophotometrically. RNA integrity was confirmed by the Agilent 2100 electrophoresis *bioanalyzer*. 1 ug of total RNA in a final volume of 20 ul was used for the synthesis of cDNA using the Superscript Vilo cDNA Synthesis Kit in accordance with the manufacturer's recommendations. cDNA was diluted to a concentration of 2.5 ng/µl with water for real-time PCR.

The NR2A, NR2B, NR2C, NR2D, NR3A, and GAPDH mRNA transcripts were quantified using the oligonucleotide primers for real-time PCR amplification designed based on sequences published in the Harvard Primer Bank and the NCBI primer-designing tool where the sequences were blasted. GAPDH was used as the housekeeping gene. The forward and reverse primer sequences used in this study are given in Table 1.

Primers were used at 300 nM final concentration. Briefly, 5 µl of the diluted synthesized cDNA together with the appropriate primers was added to 10 ul Brilliant III UltraFast SYBR green QPCR Master mix (Agilent Technologies) to a total volume of 20 µl. A PCR reaction master mix was prepared for each primer before dispensing into 96× PCR plate cells. No-template control (NTC) reactions were also prepared for each gene. Real-time PCR was carried out using a Stratagene Mx3000P (Agilent Technologies). The cycling parameters for all genes were the following: initial denaturation at 95°C for 3 min, then 40 cycles of 95°C for 20 s, and 60°C for 20 s. All 6 transcripts were measured in each unknown sample in duplicates. Expression values were normalised to GAPDH and are reported in units of 2^−ΔCt^, where ΔCt is the difference in Ct values between the target and GAPDH transcripts in the same sample.

Data are presented as fold differences of mean normalised expression values ± standard error of mean (SEM). Differences in the relative expression of each gene in HFA were verified by applying the one-way ANOVA *Tukey's multiple comparison test* using GraphPad Prism 5 (GraphPad software, San Diego, CA, USA)). Statistical significance was accepted at P<0.05; n = 4.

### Immunocytochemistry

The method is previously described [30], [31]. Briefly, purified foetal astrocytes were grown in Permanox chamber-slides until around 70–75% confluence. Cells were then fixed with methanol-acetone (1∶1) solution. Membranous permeabilization was subsequently undertaken using 0.1% Triton-X in PBS for 10 minutes at room temperature. The cells were then washed twice with PBS and incubated in PBS 5% NGS overnight at 4°C. Dilutions of the primary antibodies were made in PBS 5% NGS. Refer to Tables 2 for list of primary and secondary antibodies. Primary and secondary antibody incubations were subsequently undertaken at 37°C for 1 hr each followed by nuclear staining using DAPI (1 µg/ml) diluted 1/500 in water. Cover slips were mounted with Fluoromount-G and the slides were then viewed under the Olympus microscope BX60. Visualisation of the cells under confocal microscopy was also undertaken using the same protocol as immunocytochemistry. Slides were subsequently read under the Olympus FV1000 Laser Scanning microscope.

**Table 2:** Summary of primary and secondary antibodies and dilutions used.

PRIMARY ANTIBODIES
Category	Antibody	Brand	Isotype	Dilution
NMDAR Subunits	NR1	Abcam	Polyclonal	1/100
	NR1*	Chemicon	Monoclonal (IgG)	1/400
	NR2A/B*	Chemicon	Monoclonal (IgG)	1/400
	NR2A	Chemicon	Monoclonal (IgG)	1/50
	NR2B	Chemicon	Polyclonal	1/100
	NR2C	Novus	Polyclonal	1/100
	NR2D	Chemicon	Monoclonal (IgG)	1/50
	NR3A	Upstate	Polyclonal	1/100
	NR3B	Upstate	Polyclonal	1/100
Astrocytic Markers	Vimentin	BD Biosciences	Monoclonal (IgG)	1/200
	GFAP*	Novocastra	Monoclonal (IgG)	1/200
	GFAP*	Sigma	Polyclonal	1/200
Neuronal Markers	MAP1	Abcam	Polyclonal	1/200

SECONDARY ANTIBODIES
Antibody Type	Brand	Fluorescence	Antibody Target	Dilution
Anti-mouse	Invitrogen	Green*	Vimentin, GFAP*	1/200
Anti-rabbit	Invitrogen	Green	GFAP, MAP1	1/200
Anti-mouse	Invitrogen	Red	NR2A, NR2D	1/200
Anti-rabbit	Invitrogen	Red*	NR1, NR2B, NR2C, NR3A, NR3B, GFAP*	1/200

*Used for the double staining NMDAR-GFAP (figure 2C).

Positive controls for immunocytochemistry involved primary antibody staining of NMDARs in human primary neurons using the same protocol. Negative controls were also employed via incubations with only secondary antibodies, to detect any non-specific secondary antibody binding as well as exclude the presence of auto-fluorescent cells (data not shown).

### Calcium influx studies using fluorometry

Human foetal astrocytes were transferred into 96-well, flat-bottom plates and allowed to proliferate until confluence was reached. Astrocytic dye loading with calcium indicator was subsequently undertaken using the BD Calcium Assay Kit following the manufacturer's technical data sheet. 4 mM Probenecid was also added to the mixture followed by a 1 hr incubation period at 37°C. The loading solution was then removed and replaced with HBSS containing 50 mM glycine. Addition of selective NMDAR antagonists was undertaken 5-minutes prior to the experiment to ensure adequate diffusion time was provided to attain equilibrium. The calcium influx experiments were subsequently performed using the Fluostar Optima Fluorometer. Filter excitation and emission was set at 485 nm and 520 nm wavelength respectively. For each well, fluorescence was measured via orbital scanning of 10 locations at a 3 mm radius every 0.5 seconds. The average of these readings was recorded. Baseline fluorescence was always measured during the first 10 seconds of the experiment, which was followed by treatment with an NMDAR agonist (prepared in HBSS solution) via automated syringe injection at 100 µL/s. Fluorescent readings were subsequently taken for an additional 90 seconds. Negative controls included the injection of only HBSS solution without any agonist.

Concerning the NMDAR agonists used in the experiment, glutamate and QUIN were chosen as they are endogenous ligands found commonly in the CNS. The synthetic compound, trans-ACBD was also selected as a treatment option due to its chemical properties of being a very potent and highly selective NMDAR-specific agonist (Lanthorn et al; 1990).

### Calcium influx imaging

Human foetal astrocytes were trypsinized and transferred into 50 mm glass-bottom micro well dishes. When confluence was reached, the astrocytic growth media was removed and the cells were incubated for 1 hr at 37°C in Hepes-buffered Krebs solution containing 2 µM Fura-2 and 4 µM pluronic F-127. After dye loading, cells were washed thoroughly with Hepes-buffered Krebs solution. HBSS containing 50 mM glycine was used as the incubation medium during the experiment. Fluorescent changes caused by calcium influx were detected via a video-based imaging system in conjunction with an Olympus IX81 inverted microscope and a 20× Nikon Fluor objective lens. The emission at 520 nm was measured following excitation at 340 nm and 380 nm. Fluorescent images were recorded using the ratio of excitation by the two different wavelengths to index true changes in calcium entry. Baseline fluorescence was taken every 15 seconds for 2 minutes prior to addition of the NMDAR agonist using a manual micropipette. Fluorescent measurements were subsequently taken every 5 seconds for an additional 2 minutes following treatment. The negative control included detecting any changes in fluorescence when only HBSS solution was added.

### Lactase dehydrogenase (LDH) cytotoxicity assay

Human foetal astrocytes were trypsinized and equal volumes of cell suspension were plated into 24-well tissue culture plates. Cells were monitored regularly and allowed to reach full confluence. Selective NMDAR antagonists, MK-801, and memantine were then added in triplicates to each well at varying concentrations, followed 5 minutes later by the administration of the NMDAR agonists, QUIN and glutamate at excitotoxic levels of 500 nM and 500 µM respectively. The cells were then incubated at 37°C for 24 hours after which the media was collected and analysed for levels of LDH release.

For the LDH analysis, samples were initially diluted 1/100 and LDH activity was assayed using a standard spectrophotometric technique described by Koh and Choi [32].

LDH levels were also adjusted to take into account variations in cell number using the Bradford protein assay described by Bradford [33]. Briefly, each well was filled with 200 µl of PBS and sonication of the cells was undertaken at 20 kHz for 15 seconds in each well. These sonicated samples were collected and a set of standard protein was prepared using varying amounts of BSA. Next, 60 µl of Bradford reagent (0.01% Coomassie brilliant blue G-250, 5% ethanol, 8.5% phosphoric acid) was added to 240 µl of each sample and standard. After incubating for 30 minutes at room temperature, the samples and standards were measured for their absorbance at 595 nm using the Microplate reader 680XR (Bio-Rad; California, USA).

### Statistical analysis

Results obtained are presented as the means ± the standard error of measurement (SEM). Significant differences between results were verified using the two-tailed t-test with equal variance. Differences between treatment groups were considered significant if *p* was less than 0.05 (p<0.05).

## Results

### Detection of mRNA expression for different NMDAR sub-units using RT-PCR

**Figure 1:**
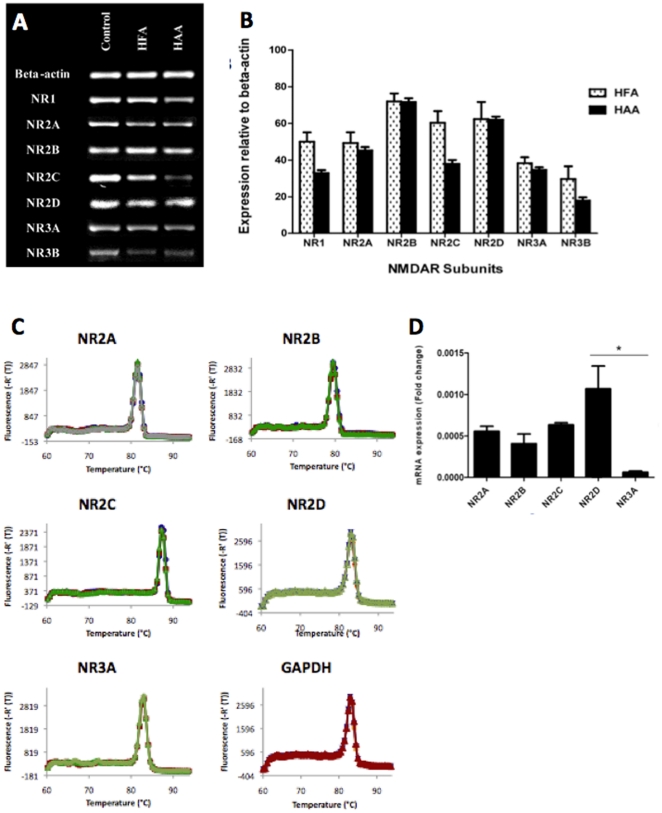
Detection of mRNA expression using PCR. End-point PCR - (1A) Agarose gel showing PCR bands for the various NMDAR subunits on human foetal astrocytes (HFA) and human adult astrocytes (HAA). Mixed foetal glial and neuronal cultures were used as positive control. (**1B**) PCR Semi-quantitative analysis of end-point PCR bands showing the relative mRNA expression levels of NMDAR subunits when compared to beta-actin in human foetal astrocytes (HFA) and human adult astrocytes (HAA). **Real-time PCR** on HFA - (**1C**) Melting curves of NR2A, NR2B, NR2C, NR2D, and NR3A transcripts. The temperature at which the rate of change of fluorescence is the greatest is defined as the melting temperature for the product. Melting curve analysis of PCR product amplified from cDNA confirms specificity of the reaction as a single peak. (**1D**) Relative mRNA expression of 5 NMDAR subunits in HFA: Histogram indicating the mRNA expression of NR2A, NR2B, NR2C, NR2D and NR3A NMDAR subunits normalised to GAPDH mRNA expression; *p<0.05 compared to NR2D and NR3A; n = 4.

End-point PCR results showed that both human foetal and adult astrocytes express the mRNA of all known NMDAR subunits including NR1, 2a, 2b, 2c, 2d, 3a, 3b (Figure 1A). Mixed brain cell cultures (including neurons, astrocytes, microglia, oligodendrocytes) were used as positive controls (first column). Semi-quantitative studies using ANOVA analysis did not reveal a significant overall difference between adult and foetal expression of the seven sub-types of NMDARs (p = 0.13) (figure 1B).

Real-time PCR has been performed human foetal astrocytes validating the above End-point PCR results and providing more quantitative data. Target genes and the housekeeping gene show high specificity as verified by a single peak in melting curve analysis (Figures 1C). Target genes and the housekeeping gene show high specificity as verified by a single peak in melting curve analysis (Figure 1C). mRNA expression of NR2A, NR2B, NR2C, NR2D, and NR3A was detected in HFA (Figure 1D). No more than 10% difference in primer efficiency between the reference gene and the target genes was obtained. NR3A mRNA expression was significantly lower compared to NR2D mRNA expression (p<0.05). GAPDH expression did not differ between all samples (data not shown).

### Detection of membranous expression of NMDAR subunits in astrocytes and neurons using immunocytochemistry and confocal microscopy

**Figure 2:**
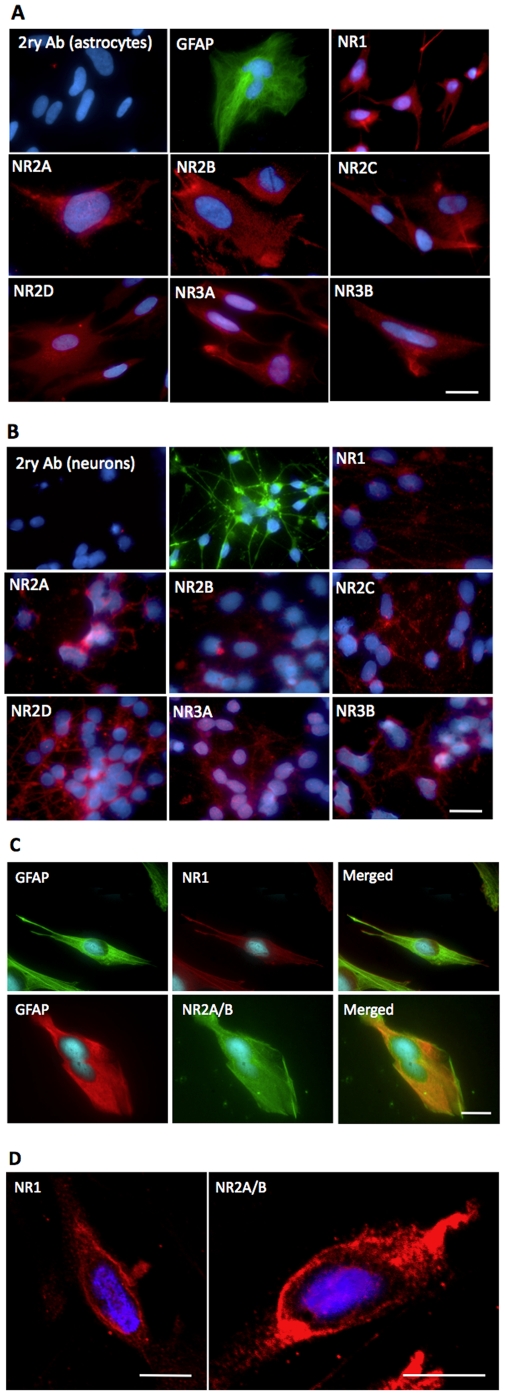
Immunocytochemical detection of NMDARs. (Scale bars = 10 µm). (**2A**) Positive controls for immunocytochemistry showing the expression of NMDARs in human foetal neurons. Immunocytochemistry results showing the presence of NR1, NR2A, NR2B, NR2C, NR2D, NR3A, NR3B in human foetal neurons at the protein level. In addition, fibrillar staining for MAP2was also undertaken to confirm that the cells were neurons. The blue DAPI staining is nuclei and the red fluorescence indicates primary antibody binding to the various NMDAR subunits. These results demonstrate that the primary antibodies for the NMDAR subunits are functional. Negative control involved incubating neurons with only secondary antibodies. (**2B**) Immunocytochemistry results showing the presence of NR1, NR2A, NR2B, NR2C, NR2D, NR3A, NR3B in human foetal astrocytes at the protein level. The blue DAPI staining marks the location of the nuclei whilst the red fluorescence shows the binding of the primary antibodies to individual NMDAR subunits. The images reveal that all NMDAR subunits are expressed in human astrocytes. Staining for GFAP was also undertaken to demonstrate the cells were of astrocytic origin. Negative control involved incubating astrocytes with only secondary antibodies (top left). (**2C**) Double staining for GFAP and NR1 and NR2A/B was performed and results show a characteristic fibrous staining of a structural protein for GFAP and fine-pigmented immunolabelling for the NMDAR subunits. (**2D**) Confocal microscopy results showing the expression of NR1 and NR2A on the surface membrane of human foetal astrocytes. The blue DAPI stain indicates the location of the nuclei whilst the red fluorescence shows binding of primary antibodies to the NMDAR subunits.

Immunocytochemistry confirmed our RT-PCR data showing that all NMDAR subunits are also expressed in human foetal astrocytes at the protein level (Figures 2B and 2C). Staining of human foetal neurons were used as positive control and revealed the presence of NMDAR expression as expected (Figure 2A). Negative controls were performed using incubations with just the secondary antibodies (Figures 2A, 2B and 2C). The presence of only blue nuclei fluorescence excludes any non-specific secondary antibody binding and also rules out the presence of auto-fluorescent cells. Incubations with an IgG irrelevant antibody as well as incubations with normal rabbit serum were also undertaken and revealed no non-specific staining. Three images were taken for each NMDAR subunit and the best image has been displayed. In addition, single staining for MAP-2 (Figure 1A) and GFAP (Figures 2A and 2C) was also undertaken to show the cells were of neuronal and astrocytic origin respectively (see also Figure S1). We then performed a double staining for GFAP and NR1 and NR2A/B. We observed a characteristic fibrous staining of a structural protein for GFAP and fine-pigmented immunolabelling for the NMDAR subunits (Figure 2C). This double staining confirms the cells expressing NMDARs are human primary astrocytes.

Confocal microscopy revealed that the majority of NR1 and NR2A subunits are located on the surface membrane of human foetal astrocytes (Figure 2C). In addition, a rim of cytoplasm mostly devoid of fluorescence can be seen separating the plasma membrane from the central lying blue-stained nucleus. From the confocal microscopy results, the detection of NMDARs on the surface membrane suggests a much higher likelihood that these ion channels are functional in astrocytes.

### Calcium influx experiments

**Figure 3:**
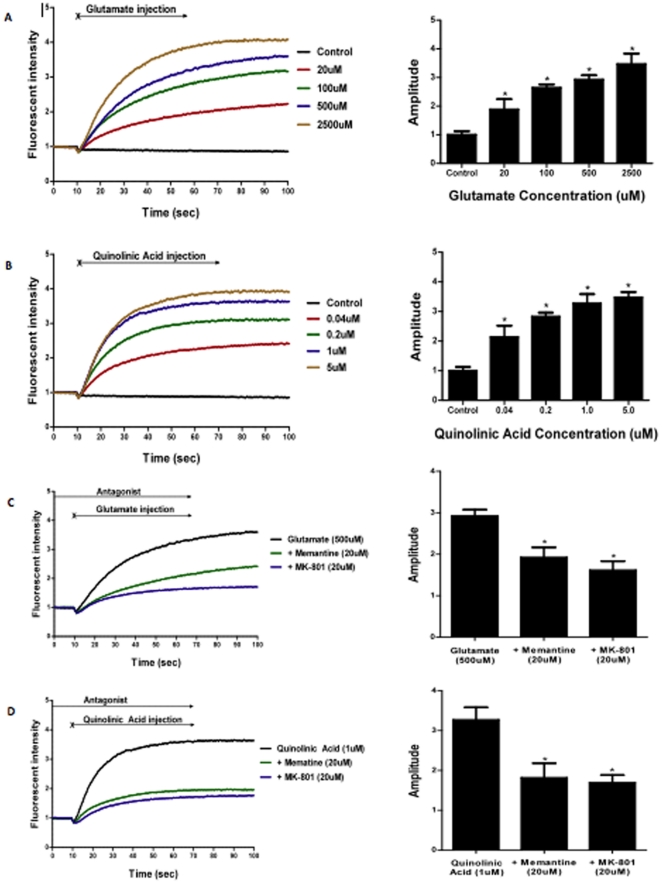
Quantification of calcium flux using fluorometry. (**A: left**) Astrocytic response to injection of different concentrations of glutamate. All results have been normalised and are representative of triplicate readings taken for each treatment. (**A: right**) Quantified amplitude of astrocytic response to glutamate by taking the average of fluorescent readings after the treatment injection at 10 seconds. Linear regression analysis shows that increasing the concentration of glutamate results in higher levels of intracellular calcium fluorescence being detected (p<0.01). (**B: left**) Astrocytic response to injection of different concentrations of quinolinic acid. The majority of intracellular calcium increase occurred within 30 seconds of agonist injection. All results have been normalised and are representative of triplicate readings taken for each treatment. (**B: right**) Quantified amplitude of astrocytic response to quinolinic acid by taking the average of fluorescent readings after the treatment injection at 10 seconds. Linear regression analysis shows that increasing the concentration of quinolinic acid results in higher levels of intracellular calcium fluorescence being detected (p<0.01). (**C: left**) Calcium influx response to glutamate when astrocytes were pre-incubated with different antagonists. In the legend, the black data set shows the level of fluorescence when only glutamate was added whilst all the other data sets show fluorescent readings when astrocytes were pre-incubated with antagonists followed by glutamate stimulation at 10 seconds. (**C: right**) Quantified amplitude of astrocytic response to glutamate when pre-incubated with NMDAR antagonists. ANOVA analysis with Dunnett's post-test shows that both treatment groups caused a calcium influx response that was statistically lower than the control (p<0.05). (**D: left**) Calcium influx response to quinolinic acid when astrocytes were pre-incubated with different antagonists. In the legend, the black data set shows the level of fluorescence when only quinolinic acid was added whilst all the other data sets show fluorescent readings when astrocytes were pre-incubated with antagonists followed by quinolinic acid stimulation at 10 seconds. (**D: right**) Quantified amplitude of astrocytic response to quinolinic acid when pre-incubated with NMDAR antagonists. ANOVA analysis with Dunnett's post test shows that both treatment groups caused a calcium influx response that was statistically lower than the control (p<0.05).

Stimulation of astrocytes using glutamate (Figure 3A) and QUIN (Figure 3B) results in calcium influx into the cells, with the intensity of the response increasing as the concentration of the NMDAR-agonist increases. The intracellular movement of calcium was significantly inhibited by pre-incubating the astrocytes with NMDAR-specific antagonists, memantine 20 µM or MK-801 20 µM for 5 min prior to stimulation. The inhibitory activity of these NMDAR-specific antagonists on glutamate and QUIN are shown in Figures 3C and 3D respectively. The glutamate concentration of 500 µM used in the experiment is well within the normal levels reached at the synaptic junction following neuronal depolarisation. QUIN 1 µM is a pathophysiological concentration found in the CNS of patients with neuroinflammatory conditions [34]. ANOVA analysis on the quantified amplitudes of astrocytic response showed that treatment with both glutamate and QUIN in the presence of specific NMDA antagonists resulted in a calcium influx response that was statistically lower than the control (p<0.05) (Figure 3).

**Figure 4:**
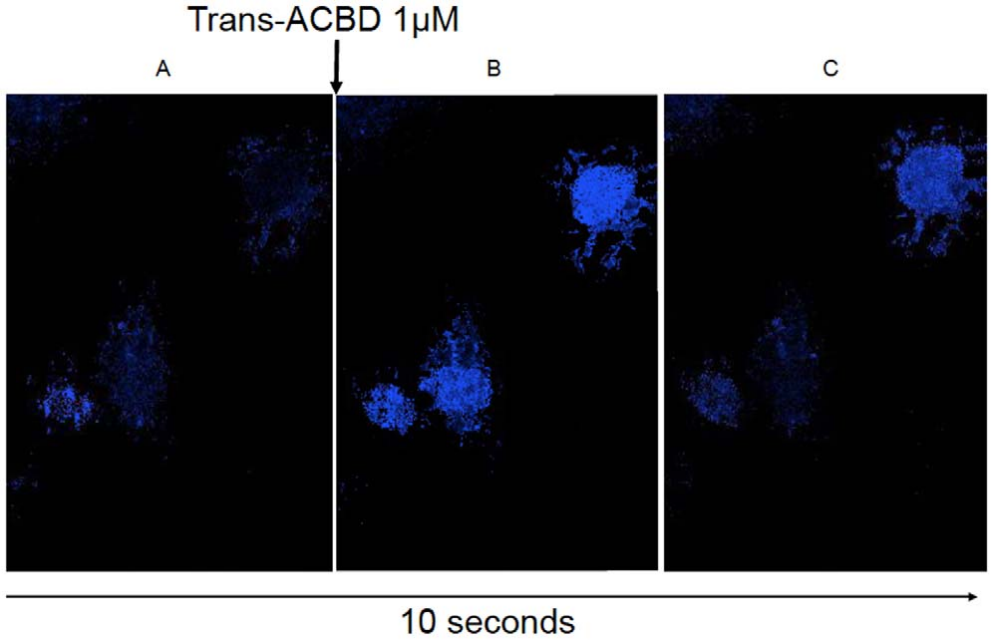
Visualization of calcium flux using fluorescent microscopy. (**A**) Fluorescent image taken before trans-ACBD treatment showing non-fluorescent astrocytes when the cells were incubating in HBSS containing 50 mM glycine. Fluorescent image taken 5 seconds (**B**) and 10 seconds (**C**) after stimulation with 1 µM trans-ACBD showing fluorescent astrocytes following treatment with a selective NMDAR agonist.

Real-time fluorescent imaging of astrocytes showed similar results, with calcium influx and subsequent cell fluorescence being detected after stimulation with trans-ACBD, which is a highly selective NMDAR agonist (Figure 4 and avi movie Video S1). One interesting observation when the experiments were being performed was that not all astrocytes responded with calcium entry following treatment with trans-ACBD. In the images taken using real-time microscopy, the cell on the bottom left did not fluoresce even after stimulation with an NMDAR agonist. This finding may reflect the heterogeneity in astrocytic NMDAR expression observed by immunocytochemistry.

### Neurotoxicity LDH assays

**Figure 5:**
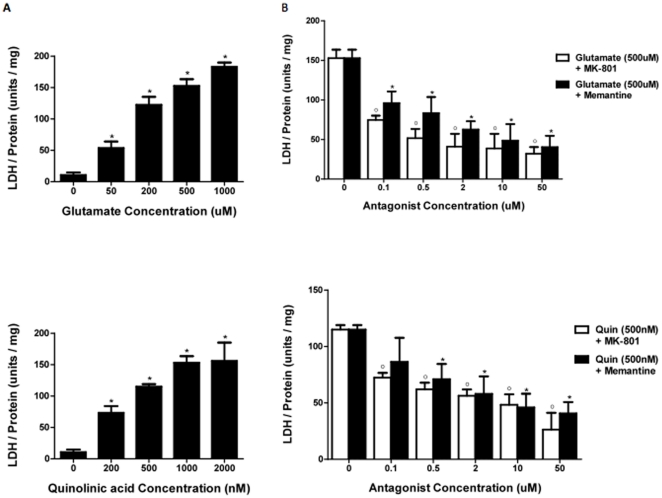
Neurotoxicity and neuroprotection assays. Different concentrations of glutamate (**A: top**) and quinolinic acid (**A: bottom**) were used to induce excitotoxic cell death in astrocytes, which were quantified by measuring LDH release after 24 hours. Protein levels were also calculated using the Bradford assay to standardise the cell numbers within each well. Significance *p<0.05 compared to control (n = 3 for each treatment group). (**B: top**) Level of LDH release after 24 hrs when astrocytes were incubated with 500 µM of glutamate and varying concentrations of different selective NMDAR antagonists. Employing two-tailed t-test analyses showed that all treatment groups of MK-801 and memantine, even at concentrations of 0.1 µM resulted in reduced LDH levels in the supernatant. Significance */^0^ p<0.05 compared to control (n = 3 for each treatment group). (**B: bottom**) Level of LDH release after 24 hrs when astrocytes were incubated with 500 nM of QUIN and varying concentrations of different selective NMDAR antagonists. Using two-tailed t-test analyses revealed that all MK-801 treatment groups resulted in reduced LDH production but only memantine concentrations above 0.5 µM were statistically significant in decreasing LDH levels. Significance */^0^ p<0.05 compared to control (n = 3 for each treatment group).

Significantly higher levels of LDH were detected in the culture supernatant following astrocyte stimulation with QUIN and glutamate (Figure 5A), reflecting increased cell death. Linear regression analysis showed that increasing concentrations of both NMDAR agonists resulted in higher levels of LDH release; glutamate (p<0.05), QUIN (p<0.05). A two-tailed t-test also revealed that the different levels of LDH release were statistically significant when treatment with glutamate (50 µM) and QUIN (200 nM) was compared to control. The data were also analysed to determine the agonist concentrations that would be most suitable for subsequent experiments involving the addition of selective NMDAR antagonists. The criteria for an appropriate excitotoxic agonist concentration included ensuring sufficient amount of cell death would occur whilst trying to maintain at least some viable astrocytes after treatment. Using the above graphs as well as observations of cell morphology after the excitotoxic incubation period, the QUIN concentration of 500 nM and glutamate concentration of 500 µM were chosen.

Pre-incubation of the astrocytes with MK-801 and memantine prior to incubation with NMDAR agonists was able to reduce LDH release, suggesting that the excitotoxic effects of both QUIN and glutamate act at least partially via the NMDAR pathway (Figure 5B). Linear regression analysis showed that both antagonists were individually effective at reducing astrocyte LDH release when added in the presence of excitotoxic concentrations of glutamate (MK-801 p<0.05; memantine p<0.01) and QUIN (MK-801 p<0.01; memantine p<0.01). Linear regression analysis with dummy variables did not show any statistical difference in the inhibitory activities of MK-801 or memantine under either conditions of excitotoxic glutamate or QUIN.

## Discussion

Astrocyte ionotropic glutamate receptors (iGluRs) and in particular NMDARs may play an important role in facilitating glial signalling in the CNS. We have shown that adult and foetal human primary astrocytes express all known NMDAR subunits including NR1. NR2A, 2B, 2C, 2D and NR3A, 3B. These results are in accordance with previous immunohistochemical studies showing that NR1 and NR2A/B are expressed by the rodent [4] and human astrocytic processes [8].

We also found that astrocyte calcium concentrations can be significantly increased when stimulated with glutamate (p<0.05) and that the mechanism of calcium elevation is at least partially attributable to activation of the NMDAR pathway. These results are supported by the real-time fluorescent microscopy data, revealing the presence of calcium entry following astrocyte stimulation with a selective NMDAR agonist (Figure 3 and 4). Results from the fluorometry studies (Figure 3) also substantiate this by showing the inhibition of calcium influx following the addition of selective NMDAR antagonists (p<0.05). Furthermore, the similar NMDAR expression profiles between adult and foetal specimens suggest that studies on foetal astrocytes may be applied to adult models. We previously compared simian adult astrocytes and human foetal astrocytes for the ability to produce chemokines and chemokines receptors and we did not found any differences between adult and foetal cells [35]. However further research is required to assess any potential differences between adult and foetal NMDARs functions of individual NMDAR subunits.

The finding that NMDAR agonists such as glutamate can cause a rise in intracellular calcium levels within astrocytes may help to explain the mechanism of glial signalling within the CNS. Whilst in neurons, sodium channels are the main receptors for initiating depolarisation, it is now known that a different form of signalling occurs in glial cells, which involves the generation of calcium waves. Astrocytic α-amino-3-hydroxy-5-methyl-4-isoxazolepropionic acid receptors (AMPARs) and metabotropic glutamate receptors (mGluRs) have been suggested to fulfil this role of facilitating rises in intracellular calcium, however the results of this study reveal that NMDAR activation may also be an important mechanism for initiating calcium influx given that these ion channels traditionally have much higher calcium permeability when compared to AMPARs [10].

The glial signalling pathways triggered by this rise in intracellular calcium are uncertain. Glutamate release has been shown to occur following an increase of cytosolic calcium in astrocytes [36]. Previously supported mechanisms of astrocytic neurotransmitter efflux were centred upon cytosolic calcium release from internal stores through the IP_3_ pathway following the activation of G-protein coupled mGluRs [36]. This study however, suggests an alternative pathway involving NMDAR activation to achieve astrocyte intracellular calcium elevation, subsequently facilitating extracellular glutamate release. Nonetheless, it is likely that both mGluRs and iGluRs are involved in glial signalling and further research would be required to determine any unique roles of each subtype.

Whilst physiological concentrations of glutamatergic neurotransmitters may be involved in glial communication in the CNS, pathological conditions can also result from prolonged exposure to excitotoxic levels of glutamate and other NMDAR agonists such as QUIN. Concerning AD, β-amyloid (Aβ) plaques are characteristic of this condition and their presence during diseased states has been well documented [37]. These neurotoxic lesions are often found surrounded by extensive areas of cellular necrosis. The mechanism by which glial excitotoxicity is caused by Aβ aggregation in the brain is still unclear [17]. One study by Guillemin [38] showed that Aβ plaques induced the expression of IDO-1 in surrounding macrophages and microglia, resulting in the increased production of QUIN from these cells [38]. This finding was supported by subsequent studies revealing that the concentration of QUIN was raised in brain sections from AD patients [17]. QUIN is also able to increase tau phosphorylation leading to the formation of neurofibrillary tangles. Memantine is able to reverse this effects and [39].

From the results of the LDH cytotoxicity experiments, the ability of selective NMDAR antagonists to reduce the level of astrocyte cell death in the presence of high concentrations of QUIN suggests that the excitotoxic effects of QUIN are at least partially mediated through their actions on glial NMDARs (Figure 3). Although it is known that QUIN is an agonist of neuronal NMDARs, this study also postulates that the neurotoxin acts via a similar mechanism in astrocytes to cause glial cell death. Furthermore, it has been shown that QUIN not only over-activates NMDARs but it can also inhibit astrocytic glutamate uptake and glutamine synthetase expression, compounding the excitotoxic environment in the CNS during neuroinflammatory conditions [24], [40]. Therefore, the results of this study suggest that astrocytic NMDARs may be responsible for the glial pathology found in AD and other neurological diseases due to their involvement in facilitating glutamate and quinolinic acid excitotoxicity.

In conclusion, the present study is the first to conclusively show the functional expression of NMDARs in human primary astrocytes. This discovery has far-reaching implications, particularly in redefining the role of astrocytes in both physiological progresses and pathological conditions.

## Supporting Information

Video S1 Real-time imaging of astrocytes showed similar results, with calcium influx and subsequent cell fluorescence being detected after stimulation with trans-ACBD.
(2.80 MB MOV)

Figure S1 Purity of primary cultures of human fetal astrocytes. GFAP immunocytochemical staining of purified human foetal primary astrocyte cultures (×400). GFAP IgG1 mAb (Novocastra) was used for this staining.
(1.35 MB TIF)
