# Supplementary figures and images for: Characterisation of the Expression of NMDA Receptors in Human Astrocytes
Source: PLoS One. 2010 Nov 30;5(11):e14123. doi: 10.1371/journal.pone.0014123 (PMC2994931; doi:10.1371/journal.pone.0014123)

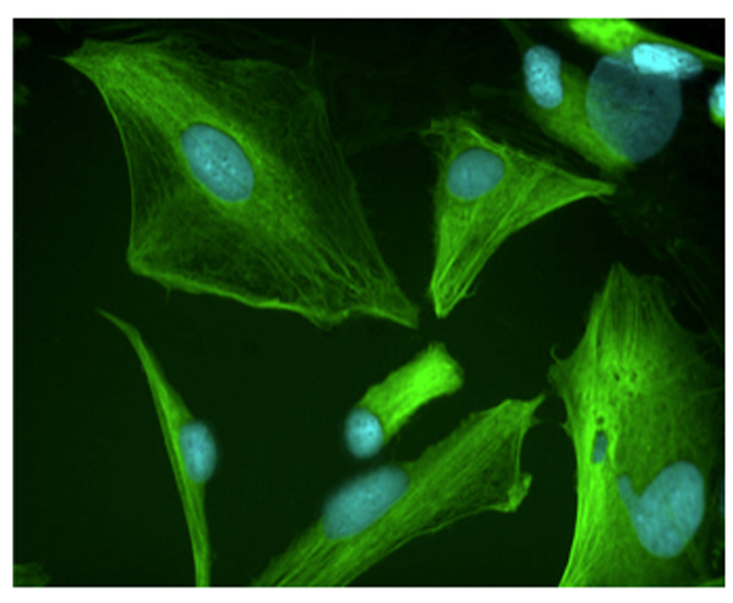

Supplement: Figure S1 — Purity of primary cultures of human fetal astrocytes. GFAP immunocytochemical staining of purified human foetal primary astrocyte cultures (×400). GFAP IgG1 mAb (Novocastra) was used for this staining. (1.35 MB TIF) [file pone.0014123.s002.tif]
